# How to hold an effective meeting

**DOI:** 10.1097/IJ9.0000000000000022

**Published:** 2017-05-24

**Authors:** Kiron Koshy, Alison Liu, Katharine Whitehurst, Buket Gundogan, Yasser Al Omran

**Affiliations:** a Brighton and Sussex University Hospital, UK; b Brighton and Sussex University Hospital, UK; c Royal Devon and Exeter Hospital, UK; d UCL Medical School, University College London, London, UK; e Royal Berkshire Hospital NHS Foundation Trust, UK

**Keywords:** Meeting, Organization, Team management, Management

## Abstract

Meetings are a common occurrence in academic and medical life. However, most of these meetings will be under-productive and inefficient uses of time. In this article, we provide valuable tips on how best to plan a meeting and get the most out of the people in attendance. This includes how to assess whether a meeting is necessary and what form this meeting should take. In addition to this, guidance is divided into before, during, and after the meeting. This guide will provide structure to your meetings and improve the output you and your team gain from them.

Around the world, millions of meetings are being held every day—most of them unproductive^1^,^2^. This wastes valuable time and effort for all involved^3^. Within Medicine, meetings are common place: from society meetings at medical school to multidisciplinary team meetings in hospitals. To increase efficiency, one must first determine whether a meeting is necessary or appropriate for the problem^4^. It is useful then to consider the events before, during, and after, to make your meeting as productive and useful as possible^5^–^7^.

## Is a meeting needed?

A meeting for meeting’s sake is unlikely to be productive. Before you call a meeting, think about whether there is any novel information or updates^8^. No new news? Consider cancelling the meeting. It is important to keep in mind that while you may have some free time in your schedule, your colleagues may have to cancel important professional or personal commitments to attend. Acknowledging the other commitments of your team will increase the efficiency of any meetings.

If there is new information, how much communication is required to find an appropriate course of action for the news? Can the update be provided in 10 to 15 minutes? If so, perhaps an email bulletin would be sufficient? Other similar problems can be solved without a meeting^9^,^10^:

- Do you need a question answered? PHONE CALL
- Are there difficult/sensitive issues? MEET ONE TO ONE

A meeting is perhaps only necessary when the subject matter(s) require at least an hour of your time, any less can usually be resolved with alternative means^11^.

## Before

Determine the structure and purpose^12^:

- What is the objective?
- Who needs to attend?
- Will an external speaker be required?
- How much time is needed?
- What preparation will help?
- What is your role?

Communicate in advance^13^:

- Develop a written agenda—assigning ownership to each item.
- Send an agenda and supporting materials in advance.
- Set expectations for attendance.
- Set context/framing for the meeting—tell people why the meeting is being held.
- Ensure appropriate rooms are booked in advance, with extra time allocated for seating rearrangement.
- If special items are required (for example audio-visual facilities) make sure that this is arranged well in advance.

For communication in advance of meetings, online surveys, for example, surveymonkey.com are commonly used tools. They can allow you to gauge expectations of topics to cover in the meeting, as well as help you to organize a convenient date for the involved parties.

On the occasions that external speakers will be required for organization meetings, it is important to contact them early and make it clear to them the reason that you have involved them in this meeting, so that they may prepare appropriately.

## During

- Start and finish on time.
- Assign a note taker and time keeper^14^.
- Provide context for meeting—again remind people why the meeting is being held.
- If people have not been properly introduced, let everyone be acquainted to encourage further productive discussion.
- Manage the discussion^2^,^15^:
  Asking for action from someone? Do it early, and be specific.
  Off-topic ideas coming up? Bring people back to the agenda—future discussion can be held on these topics.
  People talking for too long? Set time limits, move on to other topics and if there is time at the end come back to the topic.
  Want attendees to stay engaged? Use active listening strategies and keep it interactive.
  Want attendees to feel invested in the outcome? Acknowledge their mind-sets and interests verbally.
- Stick to the agenda.
- Review next steps and establish accountability^16^.
- End early.

## After

### Follow-up^17^

- Send brief notes to both meeting attendees and those absent with decisions made (or unmade) and further action items and owners.

### Debrief

- Review what worked and what did not, and note these for next time^18^.
  This can be done in person, or online survey platforms and evaluation forms can also be used.

## Conclusions

Most meetings can be improved. First, question if a meeting is even needed. Then follow the steps provided for before, during, and after the meeting to get the most out of your own and your colleague’s time.
